# Double obstacles increase gait asymmetry during obstacle crossing in people with Parkinson’s disease and healthy older adults: A pilot study

**DOI:** 10.1038/s41598-020-59266-y

**Published:** 2020-02-10

**Authors:** Diego Orcioli-Silva, Fabio Augusto Barbieri, Paulo Cezar Rocha dos Santos, Victor Spiandor Beretta, Lucas Simieli, Rodrigo Vitorio, Ellen Lirani-Silva, Lilian Teresa Bucken Gobbi

**Affiliations:** 1 São Paulo State University (UNESP), Institute of Biosciences, Posture and Gait Studies Laboratory (LEPLO), Rio Claro, Brazil; 2 Graduate Program in Movement Sciences, São Paulo State University (UNESP), Rio Claro, Brazil; 3 São Paulo State University (UNESP), Faculty of Science, Human Movement Research Laboratory (MOVI-LAB), Bauru, Brazil; 4 University of Groningen, University Medical Center Groningen, Center for Human Movement Sciences, Groningen, The Netherlands

**Keywords:** Basal ganglia, Parkinson's disease

## Abstract

Gait asymmetry during unobstructed walking in people with Parkinson’s disease (PD) has been well documented. However, under complex situations, such as environments with double obstacles, gait asymmetry remains poorly understood in PD. Therefore, the aim of this study was to analyze inter-limb asymmetry while crossing a single obstacle and double obstacles (with different distances between them) in people with PD and healthy older adults. Nineteen people with PD and 19 healthy older people performed three conditions: (i) walking with one obstacle (Single); (ii) walking with two obstacles with a 50 cm distance between them (Double-50); (iii) walking with two obstacles with a 108 cm distance between them (Double-108). The participants performed the obstacle crossing with both lower limbs. Asymmetry Index was calculated. We found that people with PD presented higher leading and trailing toe clearance asymmetry than healthy older people. In addition, participants increased asymmetry in the Double-50 compared to Single condition. It can be concluded that people with PD show higher asymmetry during obstacle crossing compared to healthy older people, independently of the number of obstacles. In addition, a challenging environment induces asymmetry during obstacle crossing in both people with PD and healthy older people.

## Introduction

Tripping over obstacles has been identified as one of the prominent causes of falls in Parkinson’s disease (PD)^1,2^. There are several PD-related factors associated with trips and falls during walking, including unsymmetrical gait parameters^3^. Gait asymmetry is characterized by a different pattern between the left and right limb extremities that reflects in unsymmetrical behavioral gait outcomes^4^. Under healthy conditions, both limbs behave similarly, indicating a high level of symmetry during gait^3^. However, pathologies such as PD result in marked spatial-temporal asymmetries during gait^3–6^, which is related to lesions in the basal ganglia region, the main area affected in PD^7,8^. In PD, the depletion of dopamine in the basal ganglia is asymmetric between cerebral hemispheres, resulting in asymmetrical dysfunction of multiple basal ganglia circuitry^9^. Because the basal ganglia provide phasic cues to several subcortical and cortical areas, e.g. the corpus callosum and supplementary motor area (SMA), uncoordinated bilateral control of gait can occur with PD^10–13^.

Substantial data have indicated that during level walking, people with PD present higher asymmetry in step length^13,14^ swing phase^3–6^, and step duration^13,15^ compared to healthy older people. Notwithstanding, behavioral outcomes underlying gait asymmetries under complex environments remain poorly understood in PD. To the best of our knowledge, to date, the effects on asymmetry of increasingly challenging models have been tested in postural control tasks^16^ and during a single obstacle avoidance^17^. Barbieri and colleagues^17^ showed that people with PD present increased gait asymmetry during single obstacle crossing compared to unobstructed walking. In addition, environments with double obstacles are typically performed during daily life activities^18,19^ and may reflect a more complex task. Therefore, understanding how asymmetry can be affected by more than one obstacle in the travel path is necessary.

Environments with double obstacles accentuate the motor impairments of PD during walking^20^. One possible explanation for this may be associated with the asymmetrical role of both legs. During obstacle crossing, one leg steps over the obstacle first (leading limb), followed by the other leg (trailing limb). Furthermore, when manipulated, the different roles (leading and trailing) between limbs can also be associated with a more highly asymmetric gait, a consequence of forcing the subjects to use the non-preferred/more affected limb to perform a different role during double obstacle avoidance. Remarkably, the adaptability of gait during obstacle negotiation seems to be related to the distance to the second obstacle^19,21^. For instance, Krell and Patla^21^ showed that foot position before the obstacle is regulated by the location of the second obstacle in the travel path in young subjects. However, it is not clear in the literature if a more complex task exacerbates the gait asymmetry and also if the position of a second obstacle affects the asymmetry. Therefore, we aimed to analyze inter-limb asymmetry while crossing a single obstacle and double obstacles (with different distances between them) in people with PD and healthy older adults. We hypothesized that (i) people with PD would present higher asymmetry during obstacle crossing than healthy older people, (ii) environments with double obstacles would increase the asymmetry of obstacle crossing parameters of people with PD, and (iii) the asymmetry would be dependent on the second obstacle location, where a short distance could represent more challenge to constrain the distance and require the participants to perform dual obstacle crossing in sequence.

## Results

Table 1 presents the characteristics of both groups. There were no significant differences between the PD group and CG group regarding the demographic characteristics. All participants successfully completed the task (without tripping).

**Table 1:** Characteristics of PD group and control group.

Demographic measure	PD group	Control group	p-value
Men/women	10/9	10/9	
Age (years)	71.53 ± 6.39	70.37 ± 6.25	0.576
Body height (cm)	161.72 ± 8.13	161.04 ± 7.12	0.784
Body mass (Kg)	67.38 ± 9.44	72.82 ± 14.86	0.187
MMSE (0–30)	27.50 ± 1.70	28.35 ± 1.30	0.111
Disease duration (years)	5.19 ± 3.03	NA	
UPDRS III (0–108)	27.05 ± 7.60	NA	
Hoehn &Yahr Scale – 1/1.5/2/2.5	1/1/9/8	NA	
Most Affected Limb – Right/Left	4/15	NA	
Levodopa equivalent dose (mg/day)	585.23 ± 400.24	NA	

Data are shown as mean ± SD. UPDRS: Unified Parkinson’s Disease Rating Scale; MMSE: Mini-Mental State Examination; NA: not applicable.

**Figure 1:**
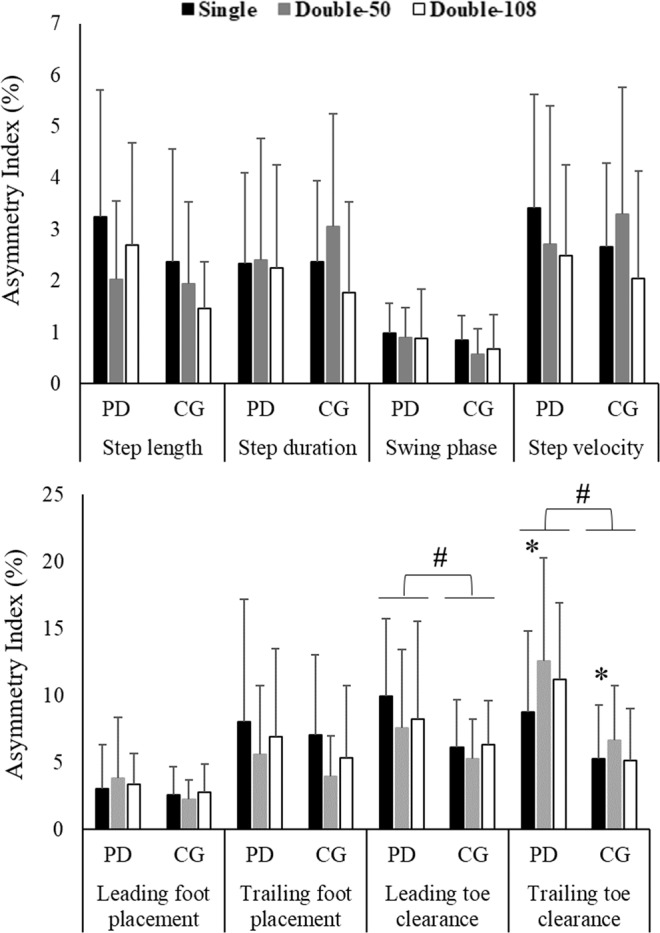
Bar graphs of means and standard deviations of Asymmetry Index of crossing step variables. *Represents the main effect of condition with significant differences in post-hoc comparisons between Single vs. Double-50 condition. ^#^Represents the main effect of group.

Table 2 and Fig. 1 show gait characteristics and Asymmetry Index of the step crossing, respectively. A main effect of group for leading (F_1,36_ = 4.425; p = 0.042, pη² = 0.109) and trailing toe clearance (F_1,36_ = 16.253; p < 0.001, pη² = 0.311) and main effect of condition for trailing toe clearance (F_2,72_ = 3,127; p = 0.05, pη² = 0.08) were identified. The PD group showed higher leading and trailing toe clearance asymmetry than the CG. In addition, participants increased asymmetry in the Double-50 compared to Single condition (p = 0.013). There was no interaction between factors.

**Table 2:** Gait characteristics of crossing step for both more affected (MAS) and less affected (LAS) limbs of PD group and both dominant limb (DL) and non-dominant limb (NDL) of control group (CG).

Gait parameters	Group	Single		Double-50		Double-108	
		MAS/NDL	LAS/DL	MAS/NDL	LAS/DL	MAS/NDL	LAS/DL
Step length (cm)	PD	63.29 ± 7.42	62.35 ± 7.59	61.58 ± 4.72	61.81 ± 3.92	61.56 ± 5.80	60.35 ± 5.52
	CG	68.54 ± 10.64	67.95 ± 8.58	64.48 ± 4.74	65.05 ± 6.80	63.24 ± 5.41	63.49 ± 5.40
Step duration (s)	PD	0.79 ± 0.12	0.78 ± 0.12	0.80 ± 0.13	0.80 ± 0.12	0.78 ± 0.12	0.77 ± 0.09
	CG	0.74 ± 0.06	0.72 ± 0.08	0.75 ± 0.09	0.73 ± 0.08	0.74 ± 0.08	0.73 ± 0.08
Swing phase (%)	PD	84.18 ± 2.43	83.21 ± 2.07	84.47 ± 1.88	83.69 ± 2.19	83.89 ± 2.18	82.89 ± 2.18
	CG	84.39 ± 2.69	84.38 ± 2.21	84.62 ± 2.55	84.09 ± 2.39	84.21 ± 2.27	83.60 ± 2.62
Step velocity (cm/s)	PD	82.77 ± 17.57	82.09 ± 16.37	78.62 ± 14.56	78.86 ± 12.80	80.79 ± 14.36	79.98 ± 12.76
	CG	94.19 ± 19.66	95.46 ± 19.24	87.66 ± 15.04	91.00 ± 16.32	86.09 ± 12.83	87.86 ± 14.32
Leading foot placement (cm)	PD	73.52 ± 10.90	75.55 ± 9.08	72.29 ± 8.92	74.50 ± 8.11	73.38 ± 9.30	75.27 ± 8.49
	CG	85.52 ± 16.43	85.09 ± 14.42	82.35 ± 15.08	82.27 ± 15.46	82.51 ± 13.27	85.08 ± 12.47
Trailing foot placement (cm)	PD	22.15 ± 5.25	23.79 ± 4.40	21.42 ± 3.86	22.69 ± 3.51	22.22 ± 5.09	23.47 ± 4.17
	CG	25.66 ± 7.58	25.38 ± 5.88	24.35 ± 4.80	24.63 ± 5.62	25.01 ± 5.41	25.45 ± 4.43
Leading toe clearance (cm)	PD	14.71 ± 4.38	15.11 ± 4.21	15.36 ± 3.17	15.45 ± 3.66	14.04 ± 3.20	14.48 ± 3.23
	CG	15.48 ± 1.93	16.34 ± 2.17	16.16 ± 2.02	16.34 ± 2.02	15.65 ± 2.74	16.29 ± 2.35
Trailing toe clearance (cm)	PD	20.90 ± 5.14	23.84 ± 6.69	21.67 ± 6.08	24.39 ± 6.77	18.18 ± 4.77	20.70 ± 5.32
	CG	22.43 ± 3.23	23.71 ± 4.19	21.04 ± 2.54	22.53 ± 3.44	21.31 ± 4.24	21.70 ± 3.83

## Discussion

We hypothesized that people with PD would present more asymmetry during obstacle crossing than healthy older people, mainly in environments with double obstacles, and this would be related to the second obstacle location. Our hypotheses were partially confirmed. Indeed, people with PD presented higher asymmetry than healthy older controls, which was observed in both leading and trailing toe clearance. Although we expected the second obstacle position to increase the asymmetry mainly in PD, the presence of double obstacles indicated a similar increase in asymmetry in both groups. Our results showed that in an environment with double obstacles positioned closer together, participants increased trailing toe clearance asymmetry compared to the single obstacle condition. Unexpectedly, no significant differences were found in temporal variables. In the following paragraphs, we discuss the higher asymmetry in the PD group, the increased gait asymmetry caused by the second obstacle in both groups, and the absence of higher asymmetry in temporal variables.

PD leads to increased asymmetry during obstacle crossing. The current results showed that people with PD present higher asymmetry in leading and trailing toe clearance than healthy older people. The obstacle crossing parameters, especially toe clearance, are treated carefully as a reduction in these variables could increase the likelihood of tripping over obstacles^22^. Previous studies have shown that people with PD present reduced toe clearance parameters compared to a control group during single obstacle^23,24^ and double obstacle conditions^20^. However, these studies did not analyze the differences between lower limb preferences and obstacle crossing role - leading and trailing limbs. The current results extend the existing knowledge by revealing that the toe clearance depends on which limb acts in each role during the obstacle crossing, higher asymmetric behavior in obstacle crossing was observed, indicating shorter distance, in MAS/NDL than LAS/DL, which could represent a higher risk of tripping. This higher asymmetry in people with PD might be a result of the disease pathophysiology. Degeneration of nigral dopaminergic neurons in basal ganglia is more predominant in one of the sides of the brain, consequently, the signs and symptoms, such as tremor, bradykinesia, and hypometria, are more evident in the contralateral side^9^, which could also be expressed in walking.

An increased asymmetry index in trailing toe clearance in the Double-50 compared to single obstacle qualitatively agrees with the idea that more challenging tasks indicate higher asymmetric gait outcomes. Previous studies have shown that challenging gait conditions, such as obstacle crossing or dual task walking, lead to increased gait asymmetry in people with PD compared to healthy older people^3,17^. However, our findings demonstrated that both people with PD and healthy controls showed higher gait asymmetry during the more challenging condition. Environments with obstacles increase the cognitive (processing and action planning), sensory (information from the environment), and motor (adaptive movements) demands to perform the task^25,26^. Thereby, due to age-related impairment in motor, cognitive, and sensory (visual, proprioceptive, and vestibular) systems^25,27,28^, locomotion in environments with obstacles becomes a challenging task, which can result in increased gait asymmetry. It is worth highlighting that the role of each limb seems to be relevant to the obstacle crossing. A decline in performance is usually observed when people with PD and older adults are forced to use the more affected/non-dominant limb to perform tasks such as standing still on a single leg^16^, an action towards a goal, and in this specific case, stepping over obstacles.

The location of the second obstacle influences the toe clearance asymmetry in both PD and healthy older adults. The current findings revealed that when obstacles are positioned closer together (50 cm distance), the trailing toe clearance asymmetry increases. Previous studies have demonstrated that both people with PD^29^ and older people^30^ prematurely transfer their gaze to the next target (obstacle) prior to completing the ongoing step, which is associated with loss of accuracy and precision of stepping movements. Therefore, we speculate that, due to the proximity between obstacles, the participants prioritize the planning of the next obstacle over the execution of ongoing steps and this may increase the asymmetry of trailing toe clearance. In addition, an alternative explanation is that the first obstacle could be affecting the crossing of the second obstacle. The 50cm-distance forces the subject to cross the second obstacle in an unstable condition. For instance, differently to crossing the first or the Double-108 condition, in which the participant is transferring the center of mass from a relatively stable to an unstable condition, the Double-50 requires the participants to cross the second obstacle immediately after the first one, in which the participant comes from one unstable condition to another unstable condition. In this latter case, the participants may direct attention to the second obstacle prematurely, which could similarly affect their safety to cross the obstacle between MAS/NDL and LAS/DL, increasing the asymmetry due to the poorer performance of MAS/NDL. This alternative explanation would be reasonable in the experimental studies which indicated that older adults and people with PD present a decline in motor performance to deal with sequential motor actions, representing a delay in planning and processing^31–33^. However, future studies should analyze gaze behavior, attention, and center of mass displacement to confirm these hypotheses.

Unexpectedly, there was an absence of higher asymmetry in temporal variables. These findings contradict the results of previous literature, which reported higher asymmetry in swing time in people with PD^3–6^. However, these previous studies analyzed gait asymmetry during unobstructed walking. A possible explanation for low asymmetry in temporal parameters is that the obstacle may have acted as an external cue. Although the presence of the second obstacle could be a distractor to crossing the first obstacle^20^, the first one may have been used as an external cue to temporally regulate both the MAS/NDL and LAS/DL during step-crossing^34,35^. Also, previous studies have shown that regulation of asymmetry may rely on cognitive function, such as attention^3,36–38^. Indeed, people with PD increase the prefrontal cortex activity during obstacle avoidance^39^. This finding may indicate that these patients increase cognitive resources, such as attention, to perform the obstacle crossing, which allows greater gait control and may imply in a low temporal asymmetry between lower limbs. Nonetheless, despite expecting temporal asymmetry in a complex environment, we manipulated spatial characteristics to constrain the distance for the participants to perform adjustments which, perhaps, required spatial instead of temporal adaptations for obstacle crossing.

Despite the novel results, this study has some limitations. For instance, it is not possible to address the neurophysiologic mechanism involved in the higher asymmetry in stride outcomes, such as cortical or muscle activity analysis. We have focused our analysis on the execution of the crossing step, as this step is considered crucial to avoid falls^26,40–42^. However, the analysis of the steps prior to the obstacles could clarify some aspects of the planning to safely obstacle crossing^43,44^. In addition, the variability analysis could be helpful to understand the moment when modulations occur and give more information about obstacle avoidance planning^24,45^. But, for a more robust analysis of variability, a larger number of analyzed steps/trials would be considered^46,47^. We have analyzed six trials per condition (three trials for each lower limb). Although relevant results, our findings need to be considered with caution and future studies should analyze more steps to confirm the current results and to calculate the gait variability. Furthermore, to enhance the range of knowledge regarding gait asymmetry in challenging tasks, further studies should consider protocols to evaluate other challenging tasks, such as adding a cognitive task concomitant to obstacle crossing or changing the obstacle height in the double obstacle condition. Another limitation could be the fixed distance between the obstacles. Previous studies have also manipulated the distance between obstacles according to step length, which may change the results^18,19^. Therefore, future studies may consider these limitations for a better understanding of the deficits caused by PD in a complex walking task.

In summary, we can conclude that, independently of the number of obstacles, people with PD show higher asymmetry in both leading and trailing toe clearance. In addition, during the double obstacle condition with obstacles positioned closer to each other, both people with PD and healthy older people increase the trailing toe clearance asymmetry compared to the single obstacle condition, showing that a more challenging environment induces asymmetry during obstacle crossing.

## Material and Methods

### Participants

The analysis with G*Power software^48^ showed that a total sample size of 38 would be necessary to obtain a power of 80% with a significance level α = 5%. Therefore, 19 people with PD and 19 healthy age-matched controls (CG) were recruited. The following exclusion criteria were used: cognitive decline (Mini-Mental State Examination - MMSE score < 24) and musculoskeletal, orthopedic, and/or visual impairments that prevent the subject from performing the required tasks. Patients were included if they were diagnosed with PD according to the UK Brain Bank criteria, classified in stages I-III of the Hoehn & Yahr Scale (H&Y)^49^, and were taking PD medication. All individuals with PD were tested in the “on state” of regular PD medication (approximately 1 h after having taken a dose). The Levodopa equivalent daily dose was calculated according to Tomlinson’s suggestions^50^. All subjects gave written informed consent, and the study was approved by the ethics committee of Sao Paulo State University at Rio Claro – Brazil (#26664014.5.0000.5465) according to the Declaration of Helsinki.

### Clinical and cognitive assess

A movement disorders specialist evaluated the clinical and cognitive aspects of participants. Global cognition was assessed using the MMSE^51^ in both PD and control groups. The PD group was clinically evaluated to determine their motor symptom severity and stage of PD using the motor portion of the Unified Parkinson’s Disease Rating Scale (UPDRS III)^52^ and the H&Y scale, respectively.

### Obstacle crossing analysis

**Figure 2:**
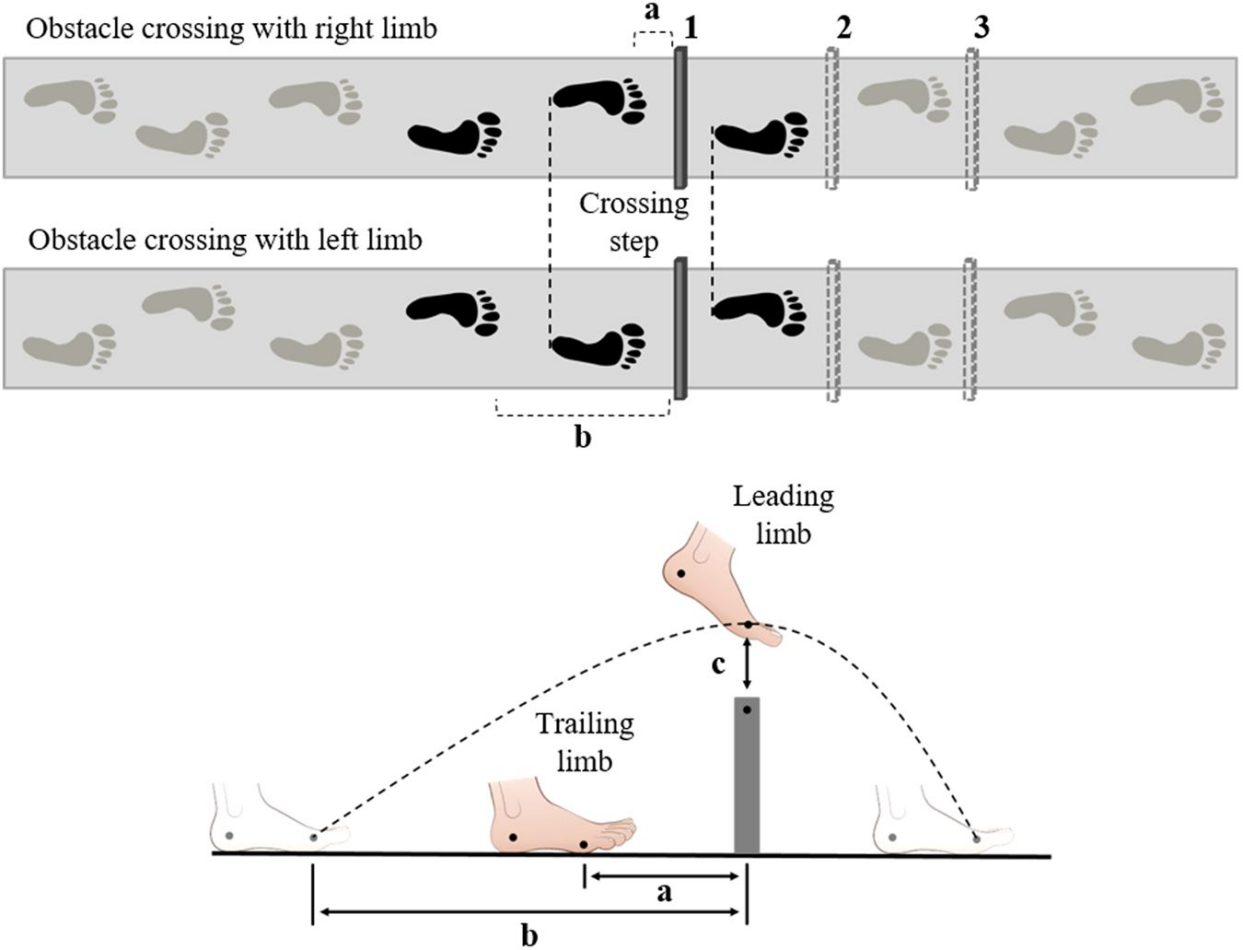
Illustration of the experimental environment. Black feet (upper figure) means the steps considered for analysis. Participants performed three trials for each limb as the leading limb. 1 - Obstacle position in Single condition and Double condition (First obstacle); 2 - Second obstacle positioned at 50 cm from the first obstacle; 3 - Second obstacle positioned at 108 cm from the first obstacle; (**a**) Trail horizontal distance before obstacle; (**b**) Lead horizontal distance before obstacle; (**c**) Toe clearance.

Participants were instructed to walk at their preferred speed on a pathway 8 m long by 0.8 m wide. Three conditions were tested: (i) walking with one obstacle (Single); (ii) walking with two obstacles with a 50 cm distance between them (Double-50); (iii) walking with two obstacles with a 108 cm distance between them (Double-108) (Fig. 2). The inter-obstacle distance was based on the approximate stride length of people with PD^20^. Six trials were performed for each condition (three trials for each limb as the leading limb). Trials were presented in a random order. The participants were also instructed to avoid contact with the obstacle, which was positioned in the middle of the pathway. The obstacles were made of foam and were 15 cm high, 3 cm thick, and 60 cm wide.

The crossing step was analyzed, meaning that in the double obstacle condition, only the first obstacle was examined (First Double) (Fig. 2). Step length, duration, velocity, and percentage of time in swing phase were calculated using the GAITRite system (CIR System, Clifton, NJ, USA), at a frequency of 200 samples/s. In addition, we used an optoelectronic tridimensional system (OPTOTRAK Certus, Northern Digital Inc., Waterloo, Ontario, Canada), at a 100 sample/s frequency, positioned in the right sagittal plane to record the outcomes related to the obstacle. Four active markers were fixed at the following anatomic points: a) 5th metatarsal and lateral face of the calcaneus of the right foot; b) 1st metatarsal and medial face of the calcaneus of the left foot. Additionally, one marker was fixed at the top edge of the obstacle. Marker trajectories were filtered with a fifth-order Butterworth low-pass filter, with a cutoff frequency of 6 Hz. Leading and trailing foot placement before the obstacle (horizontal distance from the metatarsal marker to the marker at the top edge of the obstacle) and leading and trailing toe clearance (vertical distance from the metatarsal marker to the top edge of the obstacle marker at the moment of crossing) were calculated in Matlab 7.0 (The Maths Works Inc.).

### Gait asymmetry

The sum of scores of UPDRS III items 20 to 26 was used to determine the clinically more affected side (MAS) and the opposite less affected side (less affected side: LAS). These items refer to rest tremor, action or postural tremor, rigidity, finger taps, hand movements, rapid alternating movements of the hands, and leg agility, respectively. The MAS is defined by higher scores in these items. Footedness was assessed by asking all participants to kick a ball to hit a target. The limb that each individual chose to kick the ball was considered the dominant limb (DL). The asymmetry between the MAS and LAS for people with PD and the DL and non-dominant limb (NDL) for the control group was analyzed using an Asymmetry Index (A_i_)^16,17^:

$${{\rm{A}}}_{{\rm{i}}}\,=|\begin{array}{c}\frac{{\rm{value}}\,{\rm{of}}\,{\rm{LAS}}\,{\rm{or}}\,{\rm{DL}}-{\rm{value}}\,{\rm{of}}\,{\rm{MAS}}\,{\rm{or}}\,{\rm{NDL}}}{{\rm{value}}\,{\rm{of}}\,{\rm{LAS}}\,{\rm{or}}\,{\rm{DL}}+{\rm{value}}\,{\rm{of}}\,{\rm{MAS}}\,{\rm{or}}\,{\rm{NDL}}}\end{array}|\,\times 100$$

An index value of zero indicates that there is no asymmetry. To calculate the A_i_, first, we calculated the average of each obstacle crossing parameter for each lower limb. Next, the A_i_ for each participant was calculated according to gait conditions. In walking with obstacles, each lower limb performs a specific role (leading and trailing limb), and, thus, the A_i_ was performed between corresponding steps (i.e., right and left leading limb – Fig. 2).

### Data analysis

The statistical analysis was performed with SPSS 22.0 for Windows^®^. The level of significance was set at 5% for all analysis. The normality and homogeneity of data were tested by Shapiro-Wilk and Levene’s tests, respectively. T-tests were performed to compare groups for age, body mass, and body height. MMSE score was compared between groups using the Mann-Whitney U test. A_i_ was analyzed by two-way ANOVA, with factors for group (PD group × CG) and repeated measures for condition (Single × Double-50 × Double-108). Bonferroni post hoc test was used to localize the differences when ANOVA revealed significant interactions. The partial eta squared (pη²) statistic provided estimates of the effect sizes.

## References

[1] Ashburn A, Stack E, Ballinger C, Fazakarley L, Fitton C (2008). The circumstances of falls among people with Parkinson’s disease and the use of Falls Diaries to facilitate reporting. Disabil. Rehabil..

[2] Gazibara T (2017). Near-falls in people with Parkinson’s disease: Circumstances, contributing factors and association with falling. Clin. Neurol. Neurosurg..

[3] Yogev G, Plotnik M, Peretz C, Giladi N, Hausdorff JM (2007). Gait asymmetry in patients with Parkinson’s disease and elderly fallers: When does the bilateral coordination of gait require attention?. Exp. Brain Res..

[4] Plotnik, M. & Hausdorff, J. M. The role of gait rhythmicity and bilateral coordination of stepping in the pathophysiology of freezing of gait in Parkinson’s disease. *Movement Disorders* **23** (2008). 10.1002/mds.21984 18668626

[5] Plotnik M, Giladi N, Balash Y, Peretz C, Hausdorff JM (2005). Is freezing of gait in Parkinson’s disease related to asymmetric motor function?. Ann. Neurol..

[6] Baltadjieva R, Giladi N, Gruendlinger L, Peretz C, Hausdorff JM (2006). Marked alterations in the gait timing and rhythmicity of patients with de novo Parkinson’s disease. Eur. J. Neurosci..

[7] Booij J (1997). [123I]FP-CIT SPECT shows a pronounced decline of striatal dopamine transporter labelling in early and advanced Parkinson’s disease. J. Neurol. Neurosurg. Psychiatry.

[8] Kaasinen V (2001). Personality traits and brain dopaminergic function in Parkinson’s disease. Proc. Natl. Acad. Sci. USA.

[9] Djaldetti R, Ziv I, Melamed E (2006). The mystery of motor asymmetry in Parkinson’s disease. Lancet Neurology.

[10] Fling BW (2013). Asymmetric pedunculopontine network connectivity in parkinsonian patients with freezing of gait. Brain.

[11] Debaere F (2001). Brain areas involved in interlimb coordination: A distributed network. Neuroimage.

[12] Frazzitta G, Pezzoli G, Bertotti G, Maestri R (2013). Asymmetry and freezing of gait in parkinsonian patients. J. Neurol..

[13] Fling, B. W., Curtze, C. & Horak, F. B. Gait asymmetry in people with Parkinson’s disease is linked to reduced integrity of callosal sensorimotor regions. *Front. Neurol*. **9** (2018). 10.3389/fneur.2018.00215 PMC5893803 29670573

[14] Roemmich RT (2014). Locomotor adaptation and locomotor adaptive learning in Parkinson’s disease and normal aging. Clin. Neurophysiol..

[15] Nanhoe-Mahabier W (2013). Split-belt locomotion in Parkinson’s disease with and without freezing of gait. Neuroscience.

[16] Beretta Victor Spiandor, Gobbi Lilian Teresa Bucken, Lirani-Silva Ellen, Simieli Lucas, Orcioli-Silva Diego, Barbieri Fabio Augusto (2015). Challenging Postural Tasks Increase Asymmetry in Patients with Parkinson’s Disease. PLOS ONE.

[17] Barbieri FA (2018). Obstacle Avoidance Increases Asymmetry of Crossing Step in Individuals With Parkinson’s Disease and Neurologically Healthy Individuals. J. Mot. Behav..

[18] Lowrey CR, Watson A, Vallis LA (2007). Age-related changes in avoidance strategies when negotiating single and multiple obstacles. Exp. Brain Res..

[19] Chien, J. H., Post, J. & Siu, K. C. Effects of Aging on the Obstacle Negotiation Strategy while Stepping over Multiple Obstacles. *Sci. Rep*. **8** (2018). 10.1038/s41598-018-26807-5 PMC5988752 29872074

[20] Orcioli-Silva D (2017). Walking behavior over multiple obstacles in people with Parkinson’s disease. Gait Posture.

[21] Krell J, Patla AE (2002). The influence of multiple obstacles in the travel path on avoidance strategy. Gait Posture.

[22] Vitório R (2014). Disease severity affects obstacle crossing in people with Parkinson’s disease. Gait Posture.

[23] Orcioli-Silva D (2018). Objective measures of unobstructed walking and obstacle avoidance in Parkinson’s disease subtypes. Gait Posture.

[24] Simieli, L. *et al*. Variability of crossing phase in older people with Parkinson’s disease is dependent of obstacle height. *Sci. Rep*. **8** (2018). 10.1038/s41598-018-33312-2 PMC6173742 30291294

[25] Kovacs CR (2005). Age-related changes in gait and obstacle avoidance capabilities in older adults: A review. Journal of Applied Gerontology.

[26] Galna B, Peters A, Murphy AT, Morris ME (2009). Obstacle crossing deficits in older adults: A systematic review. Gait and Posture.

[27] Van Dieën JH, Pijnappels M, Bobbert MF (2005). Age-related intrinsic limitations in preventing a trip and regaining balance after a trip. in. Safety Science.

[28] Konczak J (2009). Proprioception and Motor Control in Parkinson’s Disease. J. Mot. Behav..

[29] Vitório R, Gobbi LTB, Lirani-Silva E, Moraes R, Almeida QJ (2016). Synchrony of gaze and stepping patterns in people with Parkinson’s disease. Behav. Brain Res..

[30] Chapman GJ, Hollands MA (2007). Evidence that older adult fallers prioritise the planning of future stepping actions over the accurate execution of ongoing steps during complex locomotor tasks. Gait Posture.

[31] Melamed E, Olanow CW, Nutt JG, Lang AE (1999). Dyskinesias Assessment Workshop: Reports from the working groups. Mov. Disord..

[32] Avanzino, L., Pelosin, E., Martino, D. & Abbruzzese, G. Motor Timing Deficits in Sequential Movements in Parkinson Disease Are Related to Action Planning: A Motor Imagery Study. *Plos One* **8** (2013). 10.1371/journal.pone.0075454 PMC3781049 24086534

[33] Stöckel, T., Wunsch, K. & Hughes, C. M. L. Age-related decline in anticipatory motor planning and its relation to cognitive and motor skill proficiency. *Front. Aging Neurosci*. **9** (2017). 10.3389/fnagi.2017.00283 PMC5591340 28928653

[34] Azulay JP, Mesure S, Blin O (2006). Influence of visual cues on gait in Parkinson’s disease: Contribution to attention or sensory dependence?. J. Neurol. Sci..

[35] Vitório R (2014). Visual cues and gait improvement in Parkinson’s disease: Which piece of information is really important?. Neuroscience.

[36] Yogev G (2005). Dual tasking, gait rhythmicity, and Parkinson’s disease: Which aspects of gait are attention demanding?. Eur. J. Neurosci..

[37] Woollacott M, Shumway-Cook A (2002). Attention and the control of posture and gait: A review of an emerging area of research. Gait and Posture.

[38] O’Shea S, Morris ME, Iansek R (2002). Dual task interference during gait in people with Parkinson disease: effects of motor versus cognitive secondary tasks. Phys. Ther..

[39] Maidan I (2016). The Role of the Frontal Lobe in Complex Walking among Patients with Parkinson’s Disease and Healthy Older Adults: An fNIRS Study. Neurorehabil. Neural Repair.

[40] Galna B, Murphy AT, Morris ME (2010). Obstacle crossing in people with Parkinson’s disease: Foot clearance and spatiotemporal deficits. Hum. Mov. Sci..

[41] Stegemöller EL (2012). Postural Instability and Gait Impairment During Obstacle Crossing in Parkinson’s Disease. Arch. Phys. Med. Rehabil..

[42] da Conceição NR, de Sousa PN, Pereira MP, Gobbi LTB, Vitório R (2019). Utility of center of pressure measures during obstacle crossing in prediction of fall risk in people with Parkinson’s disease. Hum. Mov. Sci..

[43] Pieruccini-Faria F, Jones JA, Almeida QJ (2016). Insight into dopamine-dependent planning deficits in Parkinson’s disease: A sharing of cognitive & sensory resources. Neuroscience.

[44] Pieruccini-Faria, F., Ehgoetz Martens, K. A., Silveira, C. R. A., Jones, J. A. & Almeida, Q. J. Interactions between cognitive and sensory load while planning and controlling complex gait adaptations in Parkinson’s disease. *BMC Neurol*. **14** (2014). 10.1186/s12883-014-0250-8 PMC4302136 25528474

[45] Simieli Lucas, Gobbi Lilian Teresa Bucken, Orcioli-Silva Diego, Beretta Victor Spiandor, Santos Paulo Cezar Rocha, Baptista André Macari, Barbieri Fabio Augusto (2017). The variability of the steps preceding obstacle avoidance (approach phase) is dependent on the height of the obstacle in people with Parkinson's disease. PLOS ONE.

[46] Galna B, Lord S, Rochester L (2013). Is gait variability reliable in older adults and Parkinson’s disease? Towards an optimal testing protocol. Gait Posture.

[47] Kroneberg D (2019). Less Is More – Estimation of the Number of Strides Required to Assess Gait Variability in Spatially Confined Settings. Front. Aging Neurosci..

[48] Faul F, Erdfelder E, Lang AG, Buchner A (2007). G*Power 3: A flexible statistical power analysis program for the social, behavioral, and biomedical sciences. in. Behavior Research Methods.

[49] Hoehn, M. M. & Yahr, M. D. Parkinsonism: onset, progression, and mortality. 1967. *Neurology* **50**, 318 and 16 pages following (1998). 10.1212/wnl.50.2.318 9484345

[50] Tomlinson CL (2010). Systematic review of levodopa dose equivalency reporting in Parkinson’s disease. Mov. Disord..

[51] Brucki SMD, Nitrini R, Caramelli P, Bertolucci PHF, Okamoto IH (2003). Suggestions for utilization of the mini-mental state examination in Brazil. Arq. Neuropsiquiatr..

[52] Uitti RJ, Baba Y, Wszolek ZK, Putzke DJ (2005). Defining the Parkinson’s disease phenotype: Initial symptoms and baseline characteristics in a clinical cohort. Park. Relat. Disord..

